# Simultaneous stoma closure and type IV parastomal hernia repair after botulinum toxin and progressive pneumoperitoneum

**DOI:** 10.1093/jscr/rjad641

**Published:** 2023-12-05

**Authors:** Penélope Correia, Ana Marta Pereira, Maria Leonor Matos, Catarina Santos Rodrigues, Marta Guimarães, Mário Nora

**Affiliations:** General Surgery Department, Centro Hospitalar de Entre o Douro e Vouga, Santa Maria da Feira 4520-211, Portugal; General Surgery Department, Centro Hospitalar de Entre o Douro e Vouga, Santa Maria da Feira 4520-211, Portugal; General Surgery Department, Centro Hospitalar de Entre o Douro e Vouga, Santa Maria da Feira 4520-211, Portugal; General Surgery Department, Hospital da Horta, Horta 9900-038, Faial, Azores, Portugal; General Surgery Department, Centro Hospitalar de Entre o Douro e Vouga, Santa Maria da Feira 4520-211, Portugal; General Surgery Department, Centro Hospitalar de Entre o Douro e Vouga, Santa Maria da Feira 4520-211, Portugal

**Keywords:** parastomal hernia, loss of domain, stoma reversal, botulinum toxin, progressive pneumoperitoneum, transverse abdominal release

## Abstract

Surgery is the only treatment for parastomal hernia (PH). When possible, stoma closure is the best way to manage this type of hernia, however, whether to perform it in a single approach with abdominal wall reconstruction (AWR) is still debatable. A 58-year-old woman with a type IV PH with loss of domain was submitted to preoperative optimization [botulinum toxin type A and progressive pneumoperitoneum (PPP)], followed by simultaneous stoma closure and AWR. Hospital discharge was on the eighth day with no complications. Six months later, no clinical evidence of recurrence or other complication was observed. Large PHs are technically challenging. Stoma closure and simultaneous AWR increase surgical risk. Preoperative optimization with a combination of adjuvants (including PPP) is feasible in PH and may overcome technical complexity, even though patient selection remains the key when choosing a PH repair with synchronous stoma closure.

## Introduction

Parastomal hernia (PH) is the most common complication after stoma creation, tending to appear in the first 2 years. Incidence varies according to follow-up time, type of stoma, and diagnostic method, ranging from 50% to 78% in most series [1–4]. Up to 75% of cases are symptomatic with a significant negative impact on patient’s quality of life [2, 4, 5].

The only treatment remains surgery, despite lack of evidence for the best surgical option. Also, high morbidity and recurrence rates keep limiting surgical indication to only cases with disabling symptoms [1, 2, 5, 6]. Concomitant incisional hernias in other locations as well as consequences from stoma mobilization (soft tissue infections, incisional hernia) also hamper the decision-making process.

Complexity increases when loss of domain (LOD) is present (Sabbagh > 20% or Tanaka > 0.25 according to the International Delphi Consensus [7]), since primary myofascial closure becomes impossible or at the expense of negative consequences as respiratory distress or quaternary abdominal compartment syndrome [4, 8–11]. Meanwhile, the use of adjuvant techniques, such as botulinum toxin type A (BTA) and progressive pneumoperitoneum (PPP), to downstage complex hernias with LOD allowing myofascial closure, has been popularized in the last years [4, 8–11]. BTA, first described by Hurtado [12], proved to be a safe procedure, acting at pain receptors and causing a temporary flaccid paralysis with an increase of abdominal wall compliance [8, 9]. On the other hand, PPP, first described by Moreno [13], promotes not only muscle stretching but also increases abdominal cavity capacitance with a gradual increment of intraabdominal volume with air, which is also helpful with cardiopulmonary adaptation to higher pressures [4, 8–11].

High recurrence rates after PH correction are frequently observed even when optimal treatment is offered [6]. Therefore, when possible, the best way to manage this type of hernia is stoma closure. Whether to reconstruct the abdominal wall simultaneously with stoma closure is still debatable [14, 15]. Some authors have shown discouraging results regarding a single-stage procedure, as it led to higher complications and recurrence rate in comparison with isolated hernia repair or stoma closure [14–16]. Conclusions relative to the best way to manage a PH repair and a stoma closure are still lacking [14, 15].

## Case report

The authors report a single-stage approach of a PH repair with a colostomy closure after preoperative BTA and PPP adjuvant techniques.

A 58-year-old overweight (BMI = 27 kg/m^2^) woman with a history of arterial hypertension and hypothyroidism was submitted to Hartmann operation for acute diverticulitis Hinchey IV 2 years prior, complicated with superficial surgical site infection and wound dehiscence. PH was clinically evident 3 months after the index surgery.

**Figure 1:**
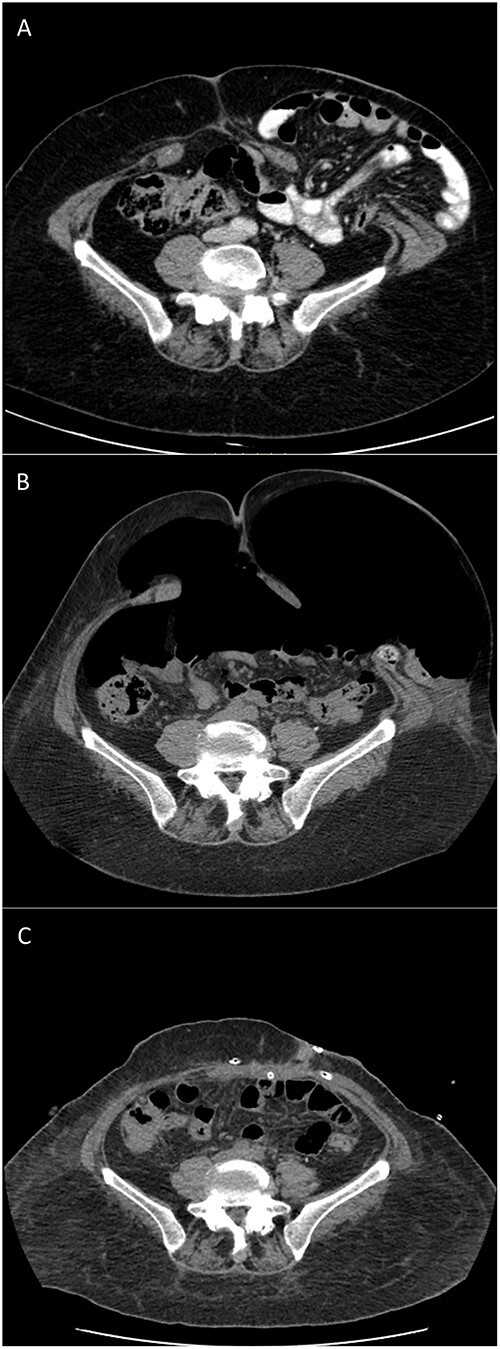
CT scans of type IV PH repair after botulinum toxin and PPP: (A) 9 months before surgery; (B) after preoperative BTA and PPP completion; and (C) 3 days postoperative.

Computed tomography (CT) scan revealed a type IV PH (according to EHS classification [17]) with LOD (Sabbagh = 20%, Tanaka = 0.25), containing small bowel without signs of ischemia or occlusion. Midline incisional hernia had a transversal diameter of 5 cm (Fig. 1A).

Patient was proposed to preoperative optimization with respiratory kinesiotherapy, adjuvant techniques (BTA and PPP), and one-stage surgery with stoma closure and hernia repair with a transverse abdominal release (TAR).

BTA was applied 4 weeks before surgery according to our institution’s protocol: 500 U of Dysport® diluted in 20 ml of 0.9% saline distributed over 3 points on each side of the lateral abdominal wall between the costal margin and the iliac crest lateral to the semilunar line. Ten days before surgery the patient was admitted for PPP. An 8 Fr pigtail was inserted by interventional radiology, and a daily insufflation of 1 l of air was performed until a total volume of 7 l was reached (Fig. 1B), limited by the patient’s intolerance for shoulder pain. Rehabilitation program, monitorization of side effects as pain, respiratory and gastrointestinal symptoms, as well as thromboembolism prophylaxis with low molecular weight heparin, compressive socks, and daily mobilization were the main goals of an inpatient procedure. No gastrointestinal complains, including colostomy retraction, were observed during the insufflation. PPP protocol was performed according to that described by Lledó *et al*. [18].

One day before surgery, a comparative CT scan demonstrated increased abdominal cavity capacitance with bowel content totally reduced (Fig. 1C).

Stoma closure with a colorectal anastomosis and PH type IV repair (midline and PHs) with a unilateral TAR (left side) were performed by placing a macroporous lightweight mesh in the retromuscular space, reaching the semilunar line on the right side. Antibiotic prophylaxis was done with 2 g cefazolin. Three drains were placed: one intraperitoneal, near the anastomosis; and two suctions drains at the retromuscular and subcutaneous planes. Intra-abdominal pressure and ventilation plateau pressure variation before incision and after closure were both close to zero. The procedure lasted 3 h and 44 min and was uneventful. The patient was admitted to the ICU, as protocolled in our institution, for total relaxation in the first 12 h and close monitoring, although no quaternary abdominal syndrome was expected according to predictive factors described by Quintela *et al*. [19].

Empirical antibiotics were started on the third postoperative day due to fever and coagulation disorders, though no objective cause was detected. CT scan was performed excluding surgical complications. The patient was discharged from ICU to the nursery on the third day and from the hospital on the eighth day. Drains were removed on the fifth day. Patient was recommended to use abdominal binder for the first postoperative month. Six months later, no clinical evidence of recurrence or other complication were observed.

## Discussion

PHs remain the Achilles tendon of the colorectal surgeon. Large PHs with LOD, associated with midline hernias, are technically challenging, with great abdominal wall disruption and myofascial retraction. Preoperative optimization with a combination of BTA and PPP, promoting abdominal volume expansion, is crucial to avoid serious postoperative complications such as abdominal compartment syndrome and hernia recurrence. Satisfactory results were described in a group of 16 patients with PHs who received the combination of BTA and PPP preoperatively, treated with the laparoscopic Sugarbaker technique [8].

Although novel techniques are evolving for PHs, such as Pauli’s operation [20], which allow simultaneous midline and PHs repair with no stoma mobilization, recurrence continues to threaten in long-term follow-ups. Stoma closure is still the best solution for PH repairs, although dilemma of losing the timing of hernia repair in a two-stage procedure or of a higher risk of postoperative complications in a one-stage procedure keeps limiting surgeons’ decision-making.

To our knowledge there are no previous reports of synchronous stoma closure and type IV PH repair after a preoperative combination of BTA and PPP.

The adjuvants used in preoperative optimization minimize the need of more disruptive techniques. A step-up approach in abdominal wall reconstruction can be offered until myofascial closure and balanced intrabdominal and ventilation pressures are achieved. This leads to less physiologic derangement, which in turn enables one-stage procedure to be done with lower overall risk, even though patient selection remains the key when choosing a PH repair with synchronous stoma closure.
